# Dynamic graph prior-guided latent adaptation for generalizable single-cell perturbation response prediction

**DOI:** 10.1093/bioinformatics/btag747

**Published:** 2026-10-12

**Authors:** Yunlong Bie, Peishan Fan, Qijin Yin, Li Xiao

**Affiliations:** School of Artificial Intelligence, Beijing University of Posts and Telecommunications, Beijing 100876, China; Zhongguancun Academy, Beijing 100094, China; School of Artificial Intelligence, Beijing University of Posts and Telecommunications, Beijing 100876, China; Zhongguancun Academy, Beijing 100094, China; School of Artificial Intelligence, Beijing University of Posts and Telecommunications, Beijing 100876, China

## Abstract

**Motivation:**

Predicting transcriptional responses to drug perturbations in unseen cellular contexts is a central challenge in single-cell perturbation modeling. This challenge creates a need for models that combine flexible state transitions with structured biological prior knowledge. Existing data-driven models often struggle to generalize across unseen cell types, while many prior-based models rely on static biological graphs without explicitly conditioning prior influence on perturbation-specific target support.

**Results:**

We propose DGP-LA, a dynamic graph-prior latent adaptation framework that refines biological priors and injects them as gated latent corrections within a cell-state-conditioned transition model. DGP-LA couples a transition backbone with perturbation-conditioned sparse graph refinement. It propagates gene-aligned target support over the refined graph and selectively injects the resulting topology-aware signal into the hidden representation. On Tahoe and NeurIPS, DGP-LA improves Pearson Delta relative to State by 8.7% and 35.8% for generalization across unseen cell types. In zero-shot drug perturbation response prediction on unseen cell types, it improves Pearson Delta relative to CRISP by 79.1% and 67.6%. The model also improves DE-gene recovery, response discrimination, and Hallmark pathway recovery, supporting adaptive latent integration of biological priors for more generalizable and biologically interpretable perturbation modeling.

**Availability:**

DGP-LA’s source code and reproducibility materials are available at https://github.com/bieyl/DGP-LA and archived on Zenodo at https://doi.org/10.5281/zenodo.22155398.

## 1 Introduction

Single-cell perturbation profiling has become a central experimental strategy for connecting interventions to cellular mechanisms. Perturb-seq profiles genetic perturbations at single-cell resolution (Dixit *et al*. 2016, Replogle *et al*. 2022), while pooled CRISPR single-cell screens enable systematic genotype–phenotype mapping (Norman *et al*. 2019). Multiplexed chemical transcriptomics extends this strategy to high-throughput pharmacological perturbation readouts (Srivatsan *et al*. 2020). However, the experimental space grows combinatorially with cell type, perturbation identity, dose, and treatment condition. Computational perturbation prediction addresses this gap by estimating responses in unmeasured cellular contexts and perturbation conditions. Such models can support virtual screening, context-specific drug response estimation, and biologically informed prioritization of candidate interventions. Effective predictors must recover both the magnitude of expression changes and the biological direction of the perturbation effect.

Perturbation responses emerge from the interaction between basal cell state and perturbation-triggered biological processes. The same intervention can induce different effects across cellular states, lineages, and disease contexts, while drug-target interactions, protein interaction neighborhoods, and downstream pathway propagation shape how those effects unfold. Models that rely only on expression data can learn statistical regularities in observed responses but often lack biological structure that supports generalization to unseen settings. Models that rely heavily on biological priors can encode prior context, yet fixed priors are often incomplete and difficult to align with cell-state-specific response dynamics. The central challenge is therefore to convert incomplete prior knowledge into adaptive support that strengthens generalization without overconstraining the learned transition.

Recent work has addressed perturbation prediction from complementary directions. Latent response and transport models learn transferable state transformations (Lotfollahi *et al*. 2019, Bunne *et al*. 2023). Compositional and chemical-response models represent perturbation covariates (Hetzel *et al*. 2022, Lotfollahi *et al*. 2023, Qi *et al*. 2024), while representation-learning and foundation-style models exploit latent cell-state structure or pretrained cellular representations (Lopez *et al*. 2018, Theodoris *et al*. 2023, Cui *et al*. 2024, Hao *et al*. 2024, Piran *et al*. 2024, Dong *et al*. 2026). State (Adduri *et al.* 2025) and CRISP (Wang *et al*. 2026) provide relevant transition and transfer references, while benchmarking studies show that gains depend on split definition, perturbation type, and cellular context (Ahlmann-Eltze *et al*. 2025, Wei *et al*. 2026). In parallel, biologically structured models have introduced graph-based message passing, perturbation-conditioned adapters, biologically informed branches, multi-knowledge-graph latent transfer, cell-type-specific co-expression priors, and pathway-guided graph convolution (Roohani *et al*. 2024, Alsulami *et al*. 2026, Bai *et al*. 2026, Guo *et al*. 2026, Wenkel *et al*. 2026, Yoon and Lee 2026). Together, these studies motivate the question of how incomplete and perturbation-dependent prior knowledge can be converted into adaptive support without sacrificing model flexibility.

Despite this progress, three limitations remain relevant to biological-prior integration. First, many methods use fixed biological graphs, although perturbation-relevant neighborhoods may vary. Second, graph-derived representations and latent state-transition representations are not automatically aligned. Third, direct prior injection may make predictions sensitive to incomplete or uncertain target annotations. Representative graph- and pathway-informed models use fixed interaction graphs, gene-level message passing, prior features, or pathway-guided components (Roohani *et al*. 2024, Alsulami *et al*. 2026, Guo *et al*. 2026, Wenkel *et al*. 2026, Yoon and Lee 2026). These designs motivate our specific focus on perturbation-conditioned graph residuals and the translation of propagated target support into a gated latent correction.

We address these limitations by formulating biological prior integration as dynamic latent adaptation. DGP-LA keeps a cell-state-conditioned backbone as the primary response predictor, but augments it with a perturbation-specific branch derived from protein–protein interaction (PPI) and drug–target interaction (DTI) evidence aligned to the highly variable gene (HVG) space. Rather than treating biological priors as fixed constraints or additional input features, DGP-LA refines them into perturbation-specific graph evidence and injects this evidence through a calibrated latent correction. This design separates the identification of prior structure relevant to a perturbation from the calibration of how strongly that structure should modify the learned transition. We evaluate DGP-LA for generalization across unseen cell types and for zero-shot drug perturbation response prediction on unseen cell types, and test whether the learned corrections align with target neighborhoods and pathway-level response programs.

## 2 Method

### 2.1 Framework overview

DGP-LA combines a data-driven transition backbone with a perturbation-conditioned prior branch, as summarized in Fig. 1a. The backbone maps control expression, perturbation identity, and dose to a hidden response transition, while the prior branch converts external target and interaction evidence into graph-derived support. Rather than imposing this support as an input feature or output constraint, DGP-LA refines target evidence in graph space (Fig. 1b) and translates the resulting support into a gated hidden-space correction (Fig. 1c). This design follows three constraints. Prior structure is adapted to each perturbation to avoid irrelevant graph neighborhoods, prior information is introduced where response transitions are represented, and prior influence is conditioned on propagated target support. The following subsections formalize these components.

**Figure 1:**
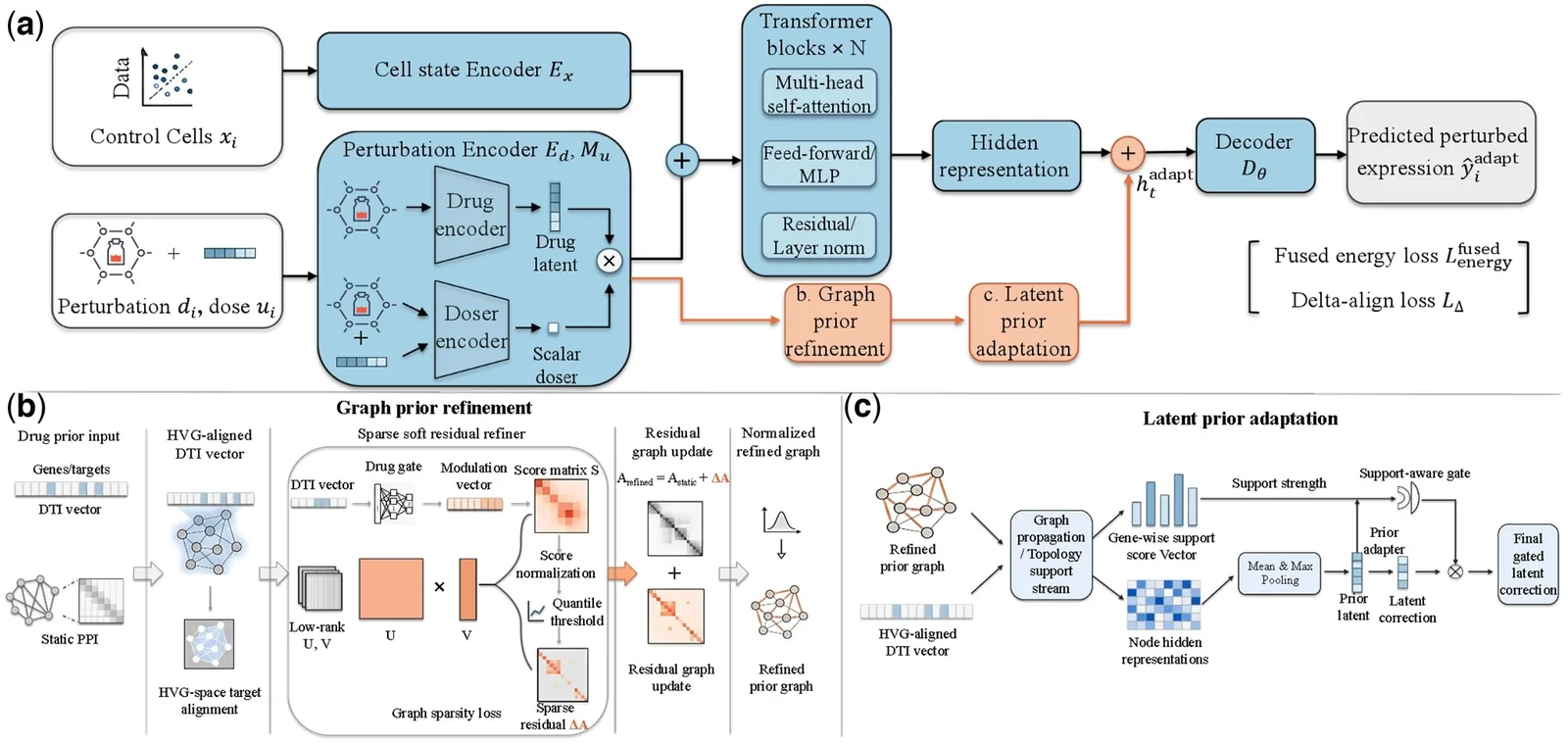
Overview of DGP-LA. (a) Cell-state-conditioned transition backbone with a gated graph-prior correction injected into the hidden response representation. (b) Perturbation-conditioned graph prior refinement from DTI and PPI inputs. (c) Topology-aware latent prior adaptation, which maps graph support into a gated hidden-space correction before final decoding.

### 2.2 Cell-state-conditioned latent transition backbone

For each sample *i*, let $x_{i}\in R^{G}$ denote the control expression profile over *G* highly variable genes and let $y_{i}\in R^{G}$ denote the observed perturbed profile. The perturbation is described by an identity $d_{i}$ and a dose feature $u_{i}$. When target annotations are available, we represent the perturbation by an HVG-aligned target vector $t_{d_{i}}\in R^{G}$, whose nonzero entries indicate genes supported by the drug-target prior. When annotations are unavailable, we set $t_{d_{i}}=0$ so that no direct target-derived graph support is introduced. We also assume a static gene interaction prior $A_{0}\in R^{G\times G}$ derived from a PPI network. In experiments, both priors are constructed once from external biological databases and kept fixed across all split-specific training runs (Section 3.2). The goal is to predict perturbed expression in generalization across unseen cell types and in zero-shot drug perturbation response prediction on unseen cell types.

The backbone serves as the primary data-driven transition learner. It maps control expression, perturbation identity, and dose into a hidden response transition before any graph-prior correction is applied. A cell-state encoder maps the control expression profile to $c_{i}=E_{x}(x_{i})\in R^{H}$, where *H* is the hidden dimension. For chemical perturbations, the identity indexes a fixed Morgan fingerprint $\phi_{d_{i}}=Q(d_{i})$, which is mapped to a drug latent $z_{d_{i}}=E_{d}(\phi_{d_{i}})$. A dose-scaling network produces $\rho_{i}=M_{u}([\phi_{d_{i}};u_{i}])$, yielding the dose-conditioned perturbation representation $p_{i}=\rho_{i}z_{d_{i}}$. Control perturbations are encoded with a zero perturbation contribution. The cell-state and perturbation representations are fused before the Transformer transition model, giving $h_{i}=T_{\theta }(c_{i}+p_{i})$. Full notation for the perturbation encoder is given in Supplementary Methods S1, available as supplementary data at *Bioinformatics* online. The graph-prior branch described below defines perturbation-level quantities $\Delta h_{d}$ and $\gamma_{d}$; for sample *i*, the corresponding latent correction and gate are $\Delta h_{d_{i}}$ and $\gamma_{d_{i}}$ from Eq. (3). The final prior-adapted hidden state and prediction are


(1) $${\hat{y}}_{i}^{\text{adapt}}=D_{\theta }(h_{i}^{\text{adapt}}+c_{i}),\;h_{i}^{\text{adapt}}=h_{i}+\gamma_{d_{i}}\Delta h_{d_{i}},$$

where $D_{\theta }$ is the decoder. This decomposition keeps the backbone as the primary data-driven response learner while allowing the graph-prior branch to supply a biologically structured correction to the hidden transition. In backbone-only ablations, the graph-prior term is removed and the prediction is decoded as $D_{\theta }(h_{i}+c_{i})$.

### 2.3 Perturbation-conditioned dynamic graph prior refinement

DGP-LA parameterizes graph refinement as a constrained perturbation-conditioned residual over a static PPI prior. This design preserves the global biological scaffold while allowing the effective neighborhood of a perturbation to adapt to its target profile.

For a perturbation with target vector $t_{d}$, we use a target-conditioned low-rank scorer to generate edge-level residual evidence. A modulation network produces $m_{d}=g_{\eta }(t_{d})\in R^{K}$, where *K* is the refinement rank, and reweights two learnable low-rank factors $U,V\in R^{G\times K}$ to form a perturbation-specific score matrix.


(2) $$S_{d}=U\mathrm{diag}(m_{d})V^{⊤}.$$

The score matrix is normalized and sparsified into a soft residual $R_{d}$, and the refined graph is obtained as $A_{d}=\mathrm{clip}(A_{0}+R_{d},0,1)$. When $t_{d}=0$, we set $R_{d}=0$ so that perturbations without target annotations do not introduce target-derived graph corrections. The shared low-rank residual retains the biological scaffold, emphasizes perturbation-relevant edges, and limits broad diffusion through unrelated regions of the network. The normalization, thresholding, and residual construction are provided in Supplementary Methods S2, available as supplementary data at *Bioinformatics* online.

### 2.4 Topology-aware latent prior adaptation

The refined graph is converted into a topology-aware support representation before entering the latent transition, ensuring that prior knowledge shapes the hidden response geometry rather than imposing hard gene-level constraints. We normalize $A_{d}$, initialize node features from the target vector $t_{d}$, and apply two GCN propagation layers to expand sparse target annotations into local multi-hop support. A learnable readout map summarizes the final node states as $\left(s_{d},r_{d})=\Omega_{\xi }(H_{d}^{\left(L\right)}\right)$, producing a gene-wise support pattern $s_{d}\in R^{G}$ and a pooled perturbation-level prior latent $r_{d}\in R^{H_{r}}$. The support pattern indicates where propagated evidence is concentrated in gene space, whereas $r_{d}$ summarizes the perturbation-specific graph response for latent adaptation. Full propagation and readout notation is given in Supplementary Methods S3, available as supplementary data at *Bioinformatics* online. The graph-derived prior latent and the backbone hidden state occupy different representational spaces. DGP-LA therefore uses a prior adapter to translate the topology-aware prior into the hidden transition space before decoding. This correction should not be applied with the same strength for every perturbation, because the amount of propagated target support varies across drugs. We therefore summarize the gene-wise support into a scalar evidence strength and combine it with the prior latent to obtain a support-aware gate.


(3) $$\Delta h_{d}=A_{\psi }(r_{d}),\;\kappa_{d}=\frac{1}{G}\sum_{g=1}^{G}|s_{d,g}|,\;\gamma_{d}=G_{\omega }([r_{d};\kappa_{d}]).$$

Here $A_{\psi }:R^{H_{r}}\to R^{H}$ is the prior adapter, $\kappa_{d}$ measures the magnitude of propagated support, [·;·] denotes concatenation, and $G_{\omega }(\cdot )$ outputs a value in [0, 1]. For a sample carrying perturbation $d_{i}$, substituting $\Delta h_{d_{i}}$ and $\gamma_{d_{i}}$ into Eq. (1) yields the final prior-adapted hidden representation and prediction. For perturbations with $t_{d}=0$, the prior branch is inactive and we set $\Delta h_{d}=0$ and $\gamma_{d}=0$, reducing the prediction to the backbone transition. Conditioning correction strength on propagated evidence allows prior influence to vary across perturbations. This controlled latent integration balances data-driven flexibility with biological guidance.

### 2.5 Training objective

The core prior-adapted objective combines three terms. The fused response term is an empirical MMD/energy distance over matched response sets, written compactly as $L_{\text{energy}}^{\text{fused}}=|S{|}^{-1}\sum_{s\in S}{\mathrm{MMD}}_{E}({\hat{Y}}_{s}^{\text{adapt}},Y_{s})$. Here S indexes matched response sets, and ${\hat{Y}}_{s}^{\text{adapt}}$ and $Y_{s}$ are the predicted and observed sets for response set *s*. The delta-alignment term matches group-level perturbation effects with a cosine loss, $L_{\Delta }=|M{|}^{-1}\sum_{m\in M}\ell_{\;\text{cos}\;}({\bar{\delta }}_{m}^{\text{pred}},{\bar{\delta }}_{m}^{\text{obs}})$. Here M indexes matched perturbation groups, $\ell_{\;\text{cos}\;}(a,b)=1-\text{cos}(a,b)$, and deltas are computed relative to control expression. The graph sparsity penalty $L_{\text{graph}}$ keeps the learned residual close to the static prior. The main objective is $L=L_{\text{energy}}^{\text{fused}}+L_{\Delta }+L_{\text{graph}}$. Full loss definitions are provided in Supplementary Methods S4, available as supplementary data at *Bioinformatics* online.

## 3 Experimental setup

### 3.1 Datasets and evaluation settings

We evaluate DGP-LA on the Tahoe (Zhang *et al.* 2026) and NeurIPS (Szałata *et al*. 2024) single-cell perturbation benchmarks. Tahoe contains 100.6 million cells from 50 cancer cell lines, with 380 drugs represented as 1138 drug-dose conditions. NeurIPS contains 2 40 059 PBMCs across six immune cell types and 147 treatment compositions. Profiles are represented in a 2000-HVG expression space, corresponding to G=2000 in the method formulation. The transition backbone uses six Transformer layers on Tahoe and four on NeurIPS, while the graph-prior design and evaluation protocol are kept unchanged. Complete model configurations and preprocessing details are provided in Supplementary Methods S5 and S6, available as supplementary data at *Bioinformatics* online.

We use two held-out settings centered on unseen cell types. In generalization across unseen cell types, all drug-perturbed cells from each held-out cell type are excluded from model training, while the perturbation vocabulary is shared with training. Control cells from held-out cell types are used only during testing as basal conditional inputs and control references. In zero-shot drug perturbation response prediction on unseen cell types, test drug identities are additionally excluded from perturbation-response training, and no test drug-dose condition appears in training, although external target annotations may remain available as prior knowledge. Tahoe results are summarized across 10 held-out cell types and NeurIPS results across four held-out immune-cell-type folds unless otherwise noted. Exact split composition and overlap statistics are provided in Supplementary Methods S7, available as supplementary data at *Bioinformatics* online.

### 3.2 Biological prior construction

For Tahoe and NeurIPS, biological priors are constructed before model training and independently of response labels. For each benchmark, we assemble a full-gene interaction scaffold $A_{0}^{\text{full}}$ and perturbation target vectors $t_{d}^{\text{full}}$ from the processed gene universe and perturbation vocabulary. This yields a PPI-derived gene-gene scaffold and a DTI-derived perturbation-target matrix, with duplicate compound-gene records collapsed before projection to the model gene space.

The PPI scaffold is derived from STRING (Szklarczyk *et al*. 2023) human protein-association evidence by retaining high-confidence mapped gene-gene interactions with combined score at least 700 and normalizing combined scores to edge weights. Drug-target annotations are aggregated from DGIdb (Freshour *et al*. 2021), DrugBank (Wishart *et al*. 2018), and ChEMBL (Mendez *et al*. 2019), and all targets are mapped to gene symbols.

At the full-gene level, the Tahoe prior contains 1 80 728 PPI edges and 6139 mapped DTI edges, covering 347 drugs with target annotations and 1746 unique target genes. The NeurIPS prior contains 1 59 015 PPI edges and 1882 mapped DTI edges, covering 128 perturbations with target annotations and 710 unique target genes. We project $A_{0}^{\text{full}}$ and $t_{d}^{\text{full}}$ onto the *G* model genes to obtain $A_{0}$ and $t_{d}$. In neighborhood analyses, direct targets are the nonzero genes in the projected DTI vector, and one-hop or two-hop genes are assigned by shortest-path distance from any direct target in the retained PPI scaffold. Projected PPI and DTI coverage is reported in Supplementary Methods S8, available as supplementary data at *Bioinformatics* online.

These priors are used only as external biological knowledge, not as response supervision. Perturbations without matched target annotations are retained with $t_{d}=0$ and receive no direct target-derived graph support. The priors are fixed before split-specific training and contain no held-out expression responses or split-specific differential-expression information. Zero-shot drug perturbation response prediction on unseen cell types should therefore be interpreted as response prediction with external target priors when available, rather than as target-free compound generalization.

### 3.3 Baselines and metrics

For generalization across unseen cell types, we compare BaseControl, BaseMLP, BioLord (Piran *et al*. 2024), scVI (Lopez *et al*. 2018), scGen (Lotfollahi *et al*. 2019), CRISP (Wang *et al*. 2026), State (Adduri *et al.* 2025), and DGP-LA. For zero-shot drug perturbation response prediction on unseen cell types, we use PRnet (Qi *et al*. 2024) and CRISP (Wang *et al*. 2026) as chemical-response references applicable to this setting.

We evaluate predictions with the open-source cell-eval package released with State (Adduri *et al.* 2025), using the perturbation and control annotations defined for each benchmark. Complete operational definitions, including the perturbation-specific value of N in Overlap@N and the normalized-rank definition of Discrimination L1, are provided in Supplementary Methods S9, available as supplementary data at *Bioinformatics* online. For figures with statistical annotations, paired t-tests are computed on matched evaluation units, and reported *q*-values are adjusted with Benjamini–Hochberg correction within each plotted comparison family.

## 4 Results

### 4.1 Generalization across unseen cell types

We first evaluate whether graph-prior adaptation supports generalization across unseen cell types. Figure 2a–f reports Tahoe and NeurIPS results using Pearson Delta, Discrimination L1, and a joint Overlap@N–MSE view. These metrics assess perturbation-effect direction, response discriminability, DE-gene recovery, and reconstruction error together.

**Figure 2:**
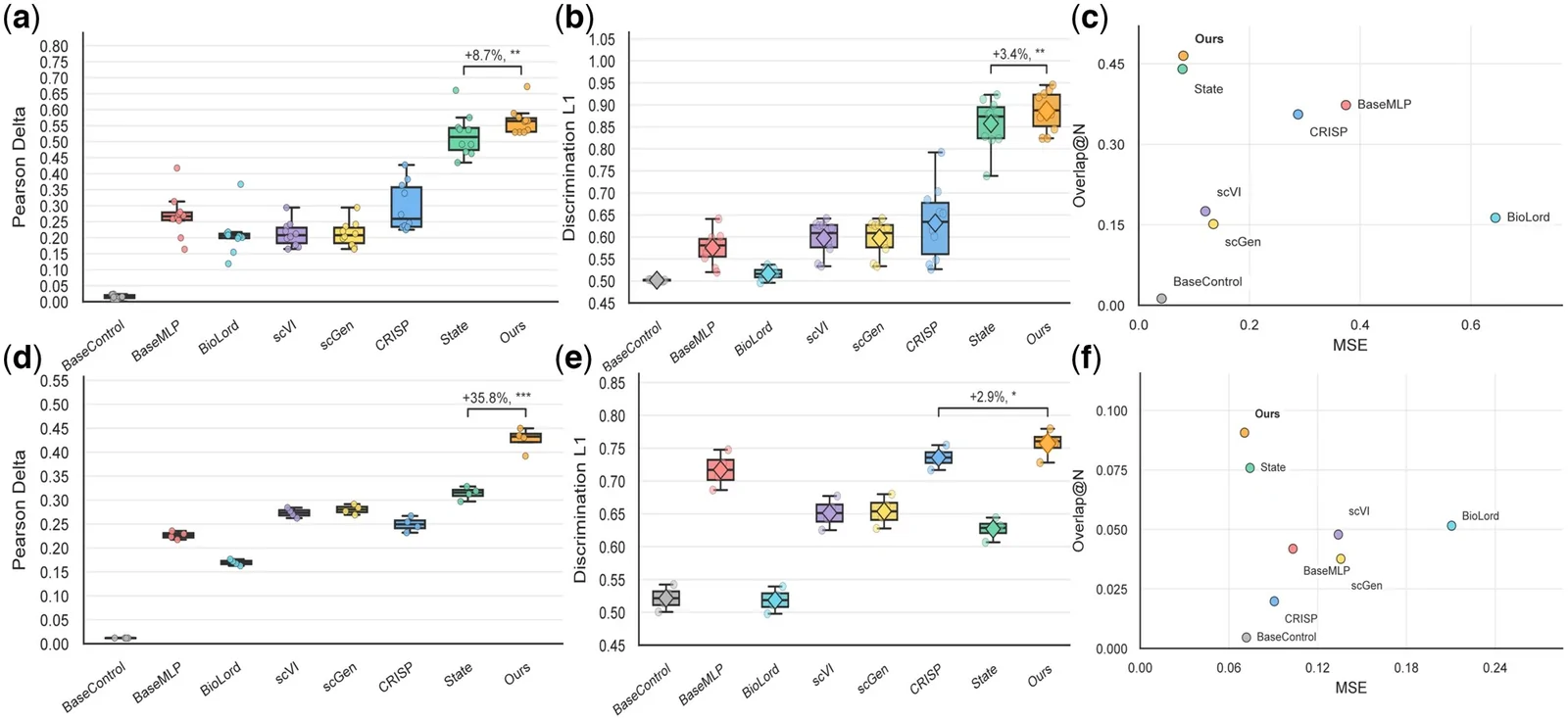
Performance across unseen cell types on Tahoe and NeurIPS. (a–c) Tahoe results across 10 held-out cell types. (d–f) NeurIPS results across four held-out immune-cell-type folds. Columns show Pearson Delta, Discrimination L1, and joint Overlap@N–MSE.

On Tahoe, DGP-LA achieves the highest Pearson Delta and Discrimination L1, as shown in Fig. 2a and b. Relative to State, the main state-transition reference in this setting, DGP-LA raises Pearson Delta by 8.7% and Discrimination L1 by 3.4%, with a smaller but consistent gain in Overlap@N (+5.6%). The joint Overlap@N–MSE view in Fig. 2c places these gains against reconstruction error, which remains comparable to State. This distinction matters because BaseControl can obtain low reconstruction error by staying close to the control state, but it fails to recover perturbation-specific DE structure. DGP-LA instead combines higher Overlap@N with low MSE, suggesting that the gain is not simply an expression-level smoothing effect.

A complementary evaluation using the Systema metrics (Viñas Torné *et al*. 2026) also favored DGP-LA over State (Table S7, available as supplementary data at *Bioinformatics* online). In a separate comparison restricted to the top 50 DEGs, DGP-LA achieved higher Pearson Delta than State and CRISP (Table S8, available as supplementary data at *Bioinformatics* online).

The corresponding NeurIPS evaluation uses held-out immune cell types rather than cancer cell lines. The pattern persists in this four-fold evaluation, with DGP-LA raising Pearson Delta by 35.8% and Discrimination L1 by 20.8% relative to State in Fig. 2d and e. The joint view in Fig. 2f shows that Overlap@N and MSE also move in the favorable direction. The agreement between Tahoe and NeurIPS suggests that the benefit is not specific to one benchmark or split definition. Instead, perturbation-conditioned graph adaptation supplies biologically structured support when the same intervention must be interpreted in a new cellular state.

### 4.2 Zero-shot drug perturbation response prediction on unseen cell types

We next evaluate zero-shot drug perturbation response prediction on unseen cell types, the more stringent generalization setting considered here. In this protocol, test drug identities and drug-perturbed cells from held-out cell types are excluded from training, and no test drug-dose condition appears in training. External target annotations may still be available as prior knowledge, as defined in the prior-construction protocol, but no held-out expression responses are used. We compare with PRnet and CRISP, two relevant chemical-response baselines, in Fig. 3a–f.

**Figure 3:**
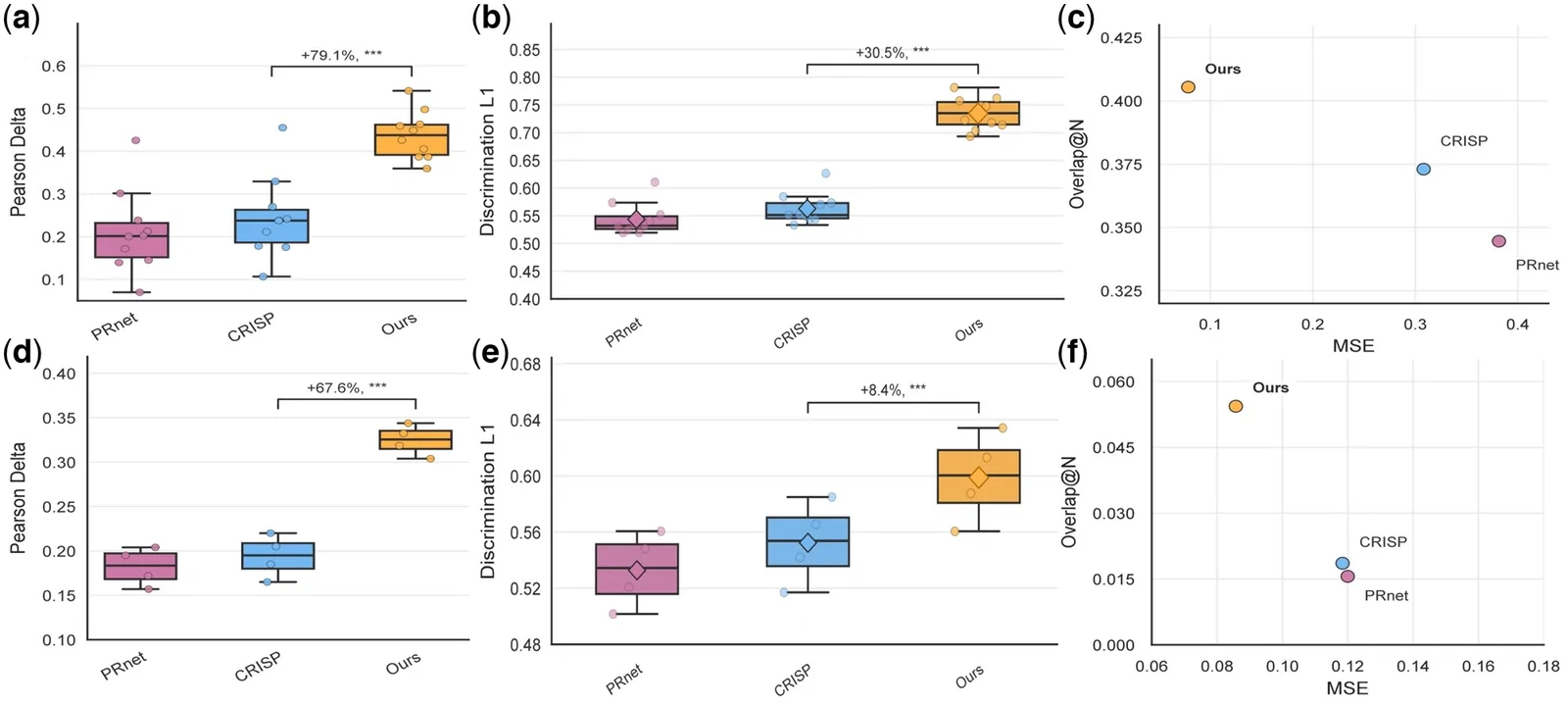
Zero-shot drug perturbation response prediction on unseen cell types. (a–c) Tahoe results across 10 held-out cell types. (d–f) NeurIPS results across four held-out immune-cell-type folds. Columns show Pearson Delta, Discrimination L1, and joint Overlap@N–MSE for PRnet, CRISP, and DGP-LA.

On Tahoe, DGP-LA improves over both baselines in the response-oriented metrics shown in Fig. 3a and b. Relative to CRISP, DGP-LA raises Pearson Delta by 79.1% and Discrimination L1 by 30.5%, with a consistent gain in Overlap@N (+10.5%). The joint view in Fig. 3c shows that MSE is reduced by 76.5%, indicating that improved biological-response recovery does not come at the cost of expression reconstruction. PRnet is comparable to CRISP in this split, but DGP-LA remains higher on the response-oriented metrics. These results suggest that the adaptive graph-prior branch is useful when the model must infer how a drug effect propagates in a cellular context whose response to that drug was not observed during training.

A similar pattern appears on NeurIPS for Pearson Delta and Discrimination L1 in Fig. 3d and e. Relative to CRISP, DGP-LA raises Pearson Delta by 67.6% and Discrimination L1 by 8.4%. The joint view in Fig. 3f shows a larger separation in DE-gene recovery, where Overlap@N improves by 616.8% and MSE decreases by 27.6%. PRnet remains below DGP-LA on response-effect correlation. Together with the Tahoe results, this comparison suggests that controlled latent corrections are helpful when drug responses must be predicted in unseen cell types.

### 4.3 Biological structure of graph-prior corrections

We next connect predictive gains to the biological structure learned by the graph branch. We analyze whether the learned correction is enriched when target priors are available, whether it concentrates near target neighborhoods, and whether it improves coordinated pathway responses. Figure 4a reports Tahoe prior-active performance, Fig. 4b examines correction magnitude by target-neighborhood distance, and Fig. 4c tests pathway-level recovery. Because only annotated perturbations can receive target-derived graph support, the prior-active subset evaluates the operating regime where the prior branch has usable biological evidence. Relative to State, DGP-LA improves Pearson Delta by 11.6% on this subset, larger than the corresponding gain on the complete Tahoe benchmark for generalization across unseen cell types. The prior-active DGP-LA result also exceeds its full-set result by 4.6%, consistent with larger gains when target support is available.

**Figure 4:**
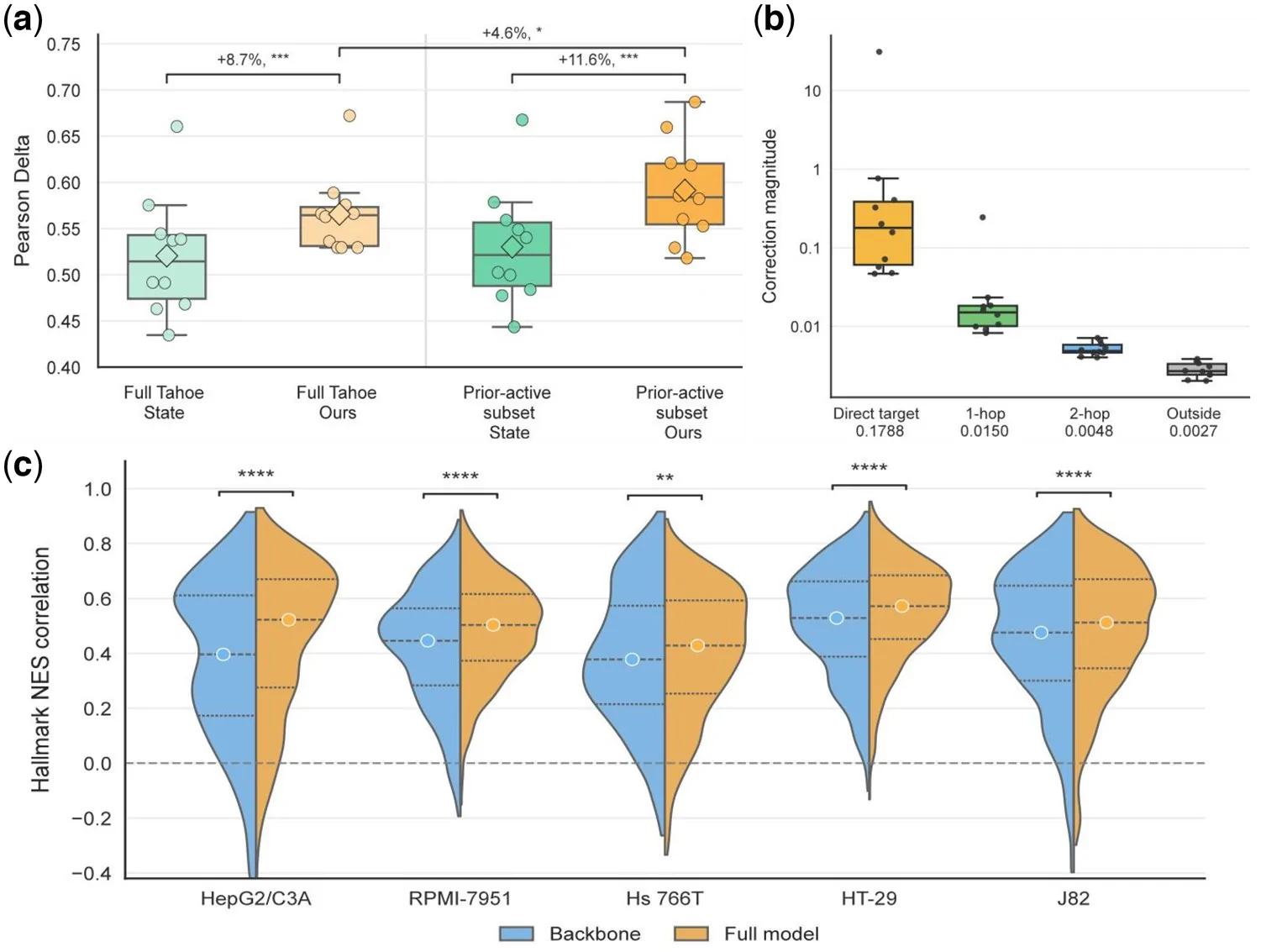
Prior-active performance, graph locality, and pathway recovery. (a) Pearson Delta on the full Tahoe benchmark and the prior-active subset. (b) Correction magnitude grouped by PPI distance from annotated targets. (c) Per-perturbation correlation between predicted and observed Hallmark NES profiles across displayed Tahoe held-out cell types.

The graph-locality analysis in Fig. 4b suggests that the learned corrections are structured by distance to annotated targets. We group genes by their PPI distance from drug targets and measure the magnitude of the full-model correction relative to the backbone. Correction magnitude is largest at direct targets and decreases with network distance, from a median of 0.1788 at direct targets to 0.0027 outside the target neighborhood. Direct-target corrections are therefore approximately 66-fold larger than the outside-neighborhood background. This distance-ordered decay supports the interpretation that the learned graph branch uses target-proximal biological support rather than applying an unstructured global adjustment.

The pathway analysis in Fig. 4c asks whether target-local corrections also improve coordinated response programs rather than only individual genes. The graph-adapted predictions better recover MSigDB Hallmark response profiles (Subramanian *et al*. 2005, Liberzon *et al*. 2015). Across the five displayed held-out cell types in Tahoe, the full model improves mean Hallmark normalized enrichment score correlation by 11.5% over the backbone. The median improvement across 3,675 matched perturbation cases is 12.3%, with the largest mean gain occurring in HepG2/C3A (+0.086). This analysis moves beyond gene-level overlap. The learned correction better preserves the direction and organization of pathway-level response programs.

The gene-level case profiles extend the aggregate locality analysis to individual drug and cell-type examples. Figure 5 shows HepG2/C3A examples, with displayed genes organized by direct target, one-hop, and two-hop PPI neighborhoods. Additional J82 and LoVo examples are provided in Supplementary Figs. S1 and S2, available as supplementary data at *Bioinformatics* online. In the displayed HepG2/C3A cases, graph prior-guided adaptation reduces prediction error relative to the observed perturbation delta for target-neighborhood genes while improving perturbation-effect correlation. These profiles show that the graph correction improves gene-level agreement in individual perturbation contexts as well as in aggregate.

**Figure 5:**
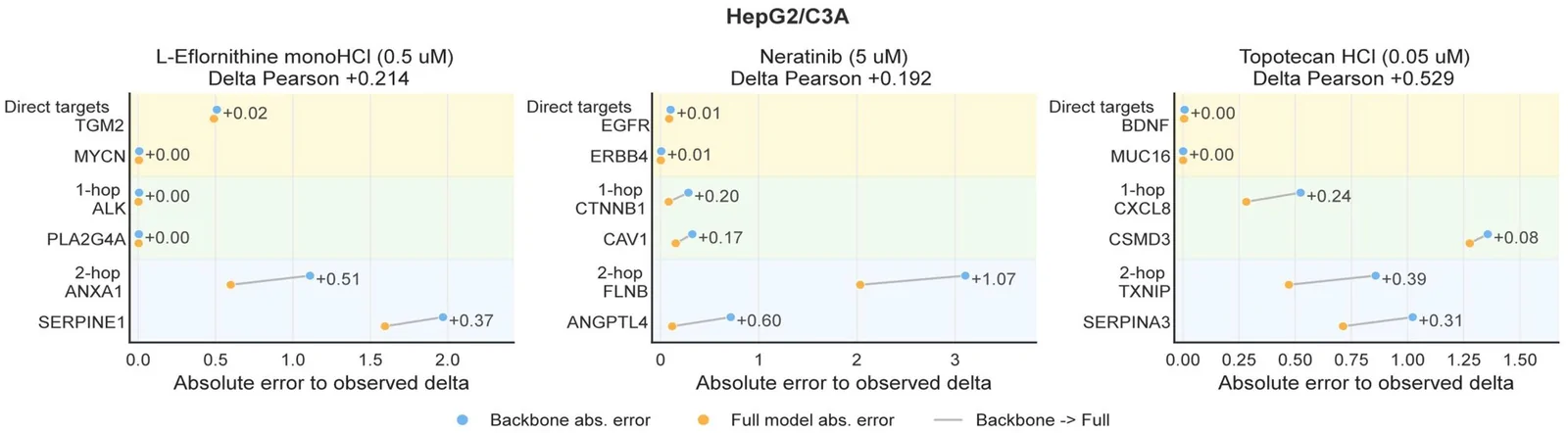
Target-neighborhood gene-level case profiles. HepG2/C3A examples are shown for three drugs. Genes are grouped by distance from annotated targets in the PPI scaffold; the horizontal axis reports prediction error relative to the observed perturbation delta.

Figure 6a–c summarize a pathway directionality case in which the graph correction improves coordinated biological programs rather than isolated genes. In HepG2/C3A treated with ivermectin at 0.5 μM, the full model changes the Hallmark pathway correlation from a negative backbone value (−0.312) to a positive value (+0.664). The recovered directions match observed cell-cycle, immune-response, xenobiotic, inflammatory, and glycolytic programs. Figure 6b and c show representative running-enrichment examples, including recovery of positive interferon gamma response and negative TNF-α signaling via NF-κB. An additional NCI-H2347 ixazomib citrate case is provided in Fig. S3, available as supplementary data at *Bioinformatics* online. Together with the aggregate neighborhood and Hallmark analyses, these cases illustrate how the adaptive prior can shift predicted responses toward target-proximal genes and coherent pathway programs.

**Figure 6:**
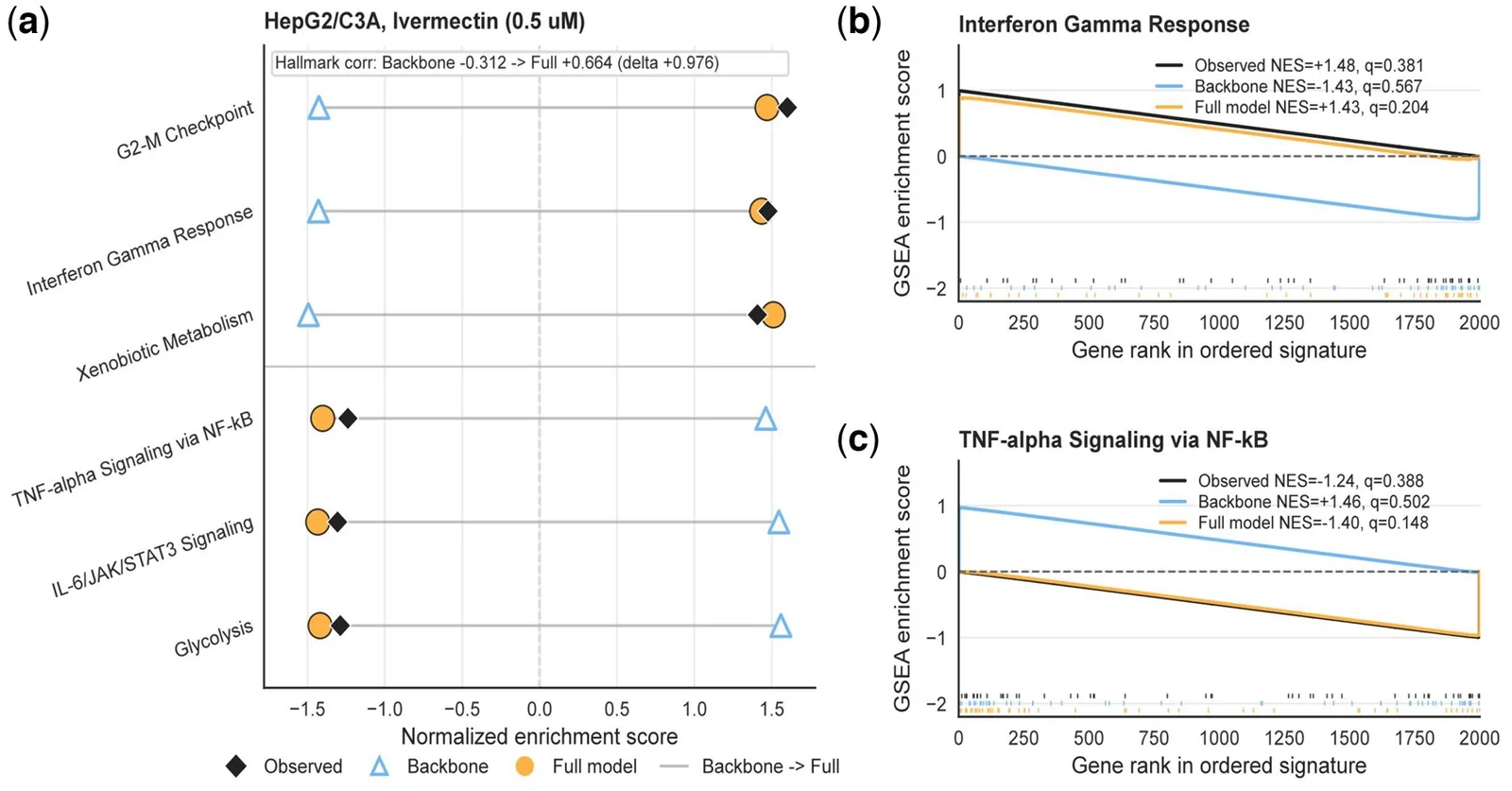
Pathway directionality recovery case. (a–c) HepG2/C3A treated with ivermectin. (a) Hallmark NES directionality. (b, c) Representative GSEA running enrichment curves.

### 4.4 Component ablation analysis

We finally ask whether the main components of DGP-LA contribute distinct functions. The Tahoe ablation study uses the same held-out cell type split design as above, with testing across 10 held-out cell types. In this separately run sweep, Backbone-only removes the graph-prior branch, whereas Full model denotes the complete DGP-LA model. Table 1 reports the three response metrics most directly targeted by graph-prior adaptation, with values shown as mean ± s.e.m. across held-out cell types.

**Table 1:** Component ablation on Tahoe.

Variant	**Pearson Delta** ↑	**Overlap@N** ↑	**Discrimination L1** ↑
Full model	**0.552** ± **0.022**	**0.451** ± **0.020**	**0.886** ± **0.021**
Backbone-only	0.520 ± 0.021	0.440 ± 0.019	0.868 ± 0.021
No delta loss	0.525 ± 0.021	0.153 ± 0.007	0.831 ± 0.020
Static PPI	0.545 ± 0.022	0.408 ± 0.018	0.863 ± 0.021
Topology-GCN 1 L	0.550 ± 0.022	0.160 ± 0.007	0.856 ± 0.021

Values are mean ± s.e.m. across 10 held-out cell types in Tahoe. Bold indicates the best value for each metric.

The ablations reveal complementary component roles. The full model improves over Backbone-only in Pearson Delta and Discrimination L1, suggesting that the prior branch improves response direction and discriminability beyond the data-driven transition backbone. Removing delta-alignment supervision produces a different failure mode. Pearson Delta changes modestly, but Overlap@N drops by 66.1%, indicating that delta alignment is important for recovering the correct differentially expressed genes rather than merely improving global correlation.

The graph-specific ablations isolate the role of prior structure. Replacing dynamic graph refinement with a static PPI prior weakens all three displayed metrics, suggesting that a fixed biological scaffold is less effective than perturbation-conditioned graph adaptation. A one-layer topology encoder leaves Pearson Delta nearly unchanged but substantially reduces Overlap@N, consistent with shallow topology propagation preserving coarse response direction while losing gene-level recovery. These distinct failure patterns support non-redundant roles for the proposed components. The model aligns perturbation deltas, refines priors dynamically, and injects topology-aware support into the latent transition space.

## 5 Discussion and conclusion

Our results support the premise that external biological priors are more effective when adapted to each perturbation and injected into the latent response model under explicit control. By learning a sparse graph residual, propagating topology-aware support, and gating the latent correction, DGP-LA addresses the mismatch between graph-space signals and learned expression-transition representations. This design bridges fixed-network biological modeling and data-driven representation learning while preserving the flexibility of the transition backbone.

The improvements span perturbation-effect direction, DE-gene recovery, response discrimination, and pathway-level coherence, with reconstruction error remaining controlled. Learned corrections concentrate near direct targets and local PPI neighborhoods, are enriched in prior-active perturbations, and improve Hallmark signature recovery. These observations indicate biological consistency rather than causal mechanism identification, which would require prospective perturbation experiments. DGP-LA is therefore suited to settings with available target annotations and downstream goals that prioritize structured perturbation effects beyond pointwise expression reconstruction. When projected target support is unavailable, the model reverts to the backbone; robustness to partial or erroneous target annotations requires separate controlled prior-perturbation experiments.

## Supplementary Material

btag747_Supplementary_Data

## Code availability

The source code and reproducibility materials for DGP-LA are available at https://github.com/bieyl/DGP-LA and archived on Zenodo at https://doi.org/10.5281/zenodo.22155398.
