# Supplementary material for: Dynamic graph prior-guided latent adaptation for generalizable single-cell perturbation response prediction
Source: Bioinformatics. 2026 Oct 12;42(10):btag747. doi: 10.1093/bioinformatics/btag747 (PMC13693364; doi:10.1093/bioinformatics/btag747)
Supplement: Supplementary file 1 [file btag747_supplementary_data.pdf]

Supplementary Information for "Dynamic Graph Prior-Guided Latent Adaptation for Generalizable Single-Cell Perturbation Response Prediction"

Supplementary Methods

S1. Backbone and perturbation encoding

For chemical perturbations, the perturbation identity indexes a fixed Morgan fingerprint $\phi_{d_i} \in \mathbb{R}^F$, and u_i records the administered dose. The perturbation encoder maps the fingerprint to a drug latent and uses a dose-scaling network to modulate that latent.

$$\begin{aligned} \phi_{d_i} &= Q(d_i), \qquad z_{d_i} = E_d(\phi_{d_i}) \in \mathbb{R}^H, \\ \rho_i &= M_u([\phi_{d_i}; u_i]) \in \mathbb{R}, \qquad p_i = \rho_i z_{d_i}. \end{aligned} \tag{S1}$$

Here $Q(\cdot)$ returns the molecular fingerprint associated with the perturbation identity, and E_d maps this fingerprint to a drug latent. The dose-scaling network M_u maps the fingerprint-dose pair to a scalar modulator ρ_i, and p_i is the resulting dose-conditioned perturbation representation. The notation $[\cdot;\cdot]$ denotes concatenation.

S2. Dynamic graph refinement

The score matrix S_d is normalized and sparsified so that high-confidence perturbation-specific corrections are retained. We compute

$$Z_d = \frac{S_d}{\mathrm{sd}(S_d) + \epsilon}, \qquad \tau_d = \mathrm{Quantile}_q(|Z_d|), \tag{S2}$$

where $\mathrm{sd}(\cdot)$ is the empirical score standard deviation, ϵ is a small numerical constant, and τ_d is the q-quantile threshold over absolute normalized scores. The sparse soft residual is

$$R_d = \alpha \tanh(\mathrm{sign}(Z_d)\,[|Z_d| - \tau_d]_+)\,, \tag{S3}$$

where $[z]_+ = \max(z, 0)$ and α is a fixed residual-scale constant. The refined prior graph is then

$$A_d = \mathrm{clip}(A_0 + R_d, 0, 1). \tag{S4}$$

Elementwise clipping preserves a valid weighted prior graph while allowing perturbation-specific edge correction.

S3. Topology-aware prior adaptation

We first normalize the refined graph as

$$\widetilde{A}_d = \Lambda_d^{-1/2} A_d \Lambda_d^{-1/2}, \qquad \Lambda_d = \mathrm{diag}(A_d \mathbf{1}) + \epsilon_A I, \tag{S5}$$

where ϵ_A keeps the degree normalization well-defined for low-degree nodes and I is the identity matrix. We initialize graph propagation by lifting each scalar target entry into a node feature with a shared target-initialization map.

$$\begin{aligned} H_{d,g}^{(0)} &= \Psi(t_{d,g}) \in \mathbb{R}^{H_g}, \quad g = 1, \ldots, G, \\ H_d^{(\ell+1)} &= \sigma\left(\widetilde{A}_d H_d^{(\ell)} W_\ell\right). \end{aligned} \tag{S6}$$

Here $\Psi : \mathbb{R} \to \mathbb{R}^{H_g}$ maps the target value of each gene to an initial graph node feature, $H_d^{(0)}$ stacks these row features across genes, and superscripts index graph-propagation layers. W_ℓ is a learnable propagation weight for layer ℓ, and $\sigma(\cdot)$ denotes a pointwise nonlinearity. We use $L = 2$ GCN propagation layers to capture local multi-hop support while limiting over-smoothing. After L propagation layers, a learnable readout map summarizes the final node states as $(s_d, r_d) = \Omega_\xi(H_d^{(L)})$, where $H_d^{(\ell)} \in \mathbb{R}^{G \times H_g}$ and H_g is the graph hidden dimension.

S4. Training objective details

Following the distributional objective used in State-style transition models (Adduri et al., 2025), we use the empirical MMD induced by the energy kernel $k(u, v) = -\|u - v\|_2$. For predicted and observed expression sets $\widehat{Y} = \{\tilde{y}_a\}_{a=1}^n$ and $Y = \{y_b\}_{b=1}^m$, the response energy distance is

$$\begin{aligned}\mathcal{E}(\widehat{Y}, Y) = \frac{2}{nm} \sum_{a=1}^{n} \sum_{b=1}^{m} \|\tilde{y}_a - y_b\|_2 - \frac{1}{n^2} \sum_{a=1}^{n} \sum_{a'=1}^{n} \|\tilde{y}_a - \tilde{y}_{a'}\|_2 \\ - \frac{1}{m^2} \sum_{b=1}^{m} \sum_{b'=1}^{m} \|y_b - y_{b'}\|_2.\end{aligned} \tag{S7}$$

For a training mini-batch, let $\mathcal{S}$ denote the collection of matched response sets, grouped by the same response condition, and let I_s be the sample indices in set $s \in \mathcal{S}$. The fused prior-adapted response loss is

$$\mathcal{L}_{\text{energy}}^{\text{fused}} = \frac{1}{|\mathcal{S}|} \sum_{s \in \mathcal{S}} \mathcal{E}\left(\{\hat{y}_i^{\text{adapt}}\}_{i \in I_s}, \{y_i\}_{i \in I_s}\right). \tag{S8}$$

Replacing $\hat{y}_i^{\text{adapt}}$ with the backbone-only prediction $D_\theta(h_i + c_i)$ gives the analogous response loss used for backbone-only training and ablation comparisons. The backbone-only version is not part of the prior-adapted objective below.

Let $\mathcal{B} = \cup_{s \in \mathcal{S}} I_s$ denote the sample indices in the mini-batch. To emphasize perturbation-induced change alongside absolute expression reconstruction, we align predicted and observed response deltas using the control input x_i as the expression-space anchor. Let $\mathcal{M}$ denote matched perturbation groups used for delta alignment. For each group $m \in \mathcal{M}$ with index set I_m, we compute

$$\bar{\delta}_m^{\text{pred}} = \frac{1}{|I_m|} \sum_{i \in I_m} (\hat{y}_i^{\text{adapt}} - x_i), \qquad \bar{\delta}_m^{\text{obs}} = \frac{1}{|I_m|} \sum_{i \in I_m} (y_i - x_i). \tag{S9}$$

The delta-alignment loss is then

$$\mathcal{L}_\Delta = \frac{1}{|\mathcal{M}|} \sum_{m \in \mathcal{M}} \left(1 - \frac{\langle \bar{\delta}_m^{\text{pred}}, \bar{\delta}_m^{\text{obs}} \rangle}{\|\bar{\delta}_m^{\text{pred}}\|_2 \, \|\bar{\delta}_m^{\text{obs}}\|_2 + \epsilon_\Delta}\right), \tag{S10}$$

where ϵ_Δ is a small numerical constant. This term encourages the model to match the direction of perturbation-induced transcriptional change, rather than only minimizing absolute expression error.

The graph residual is regularized to remain sparse so that refinement stays close to the static prior. Let $\mathcal{D}_\mathcal{B} = \{d_i : i \in \mathcal{B}, \, t_{d_i} \neq 0\}$ be the perturbations in the mini-batch with available target priors. We use

$$\mathcal{L}_{\text{graph}} = \frac{1}{|\mathcal{D}_\mathcal{B}|} \sum_{d \in \mathcal{D}_\mathcal{B}} \frac{1}{G^2} \|R_d\|_1, \tag{S11}$$

with the term set to zero when $\mathcal{D}_\mathcal{B}$ is empty.

S5. DGP-LA architecture and optimization settings

Configuration selection used the training partition of each benchmark. Architecture capacity was set once at the dataset level according to the number of training cells and drug-dose conditions and the maximum cell-set length; candidate capacities were assessed for stable optimization and feasibility at that maximum set length. The Tahoe configuration uses a hidden dimension of 1,488, six Transformer layers, and 12 attention heads for its larger training corpus and cell sets of up to 512 cells; the NeurIPS configuration uses a hidden dimension of 672, four Transformer layers, and eight attention heads for cell sets of up to 128 cells. Learning rates were retained from training-only development runs based on stable optimization of the training objective. The training budgets were fixed when the corresponding objectives had stabilized, yielding 8,000 optimization steps for Tahoe and 5,000 for NeurIPS. The resulting dataset-level configuration was then held fixed across all held-out evaluation units. Comparison methods used configurations from their respective publications or official implementations, with one configuration applied across all held-out evaluation units. Supplementary Tables S1 and S2 report these settings. The graph-refinement and topology-aware latent prior adaptation settings are shared across the two benchmarks. Component ablations retain the corresponding benchmark configuration and modify only the component under study.

Supplementary Table S1. Principal DGP-LA architecture settings.

Setting	Tahoe	NeurIPS
Expression dimension G	2,000	2,000
Morgan fingerprint dimension F	1,024	1,024
Maximum cell-set length	512	128
Transformer hidden / feed-forward dimensions	1,488 / 5,952	672 / 2,688
Transformer layers / attention heads	6 / 12	4 / 8
Drug/dose and response MLP layers / activation / dropout	4 / GELU / 0.1	4 / GELU / 0.1
Graph refinement rank K	32	32
Sparse-residual quantile q / scale α	0.999 / 1.0	0.999 / 1.0
Graph hidden dimension H_g / GCN layers L	32 / 2	32 / 2
Prior latent H_r / adapter hidden dimension	64 / 64	64 / 64
Support-aware gate hidden dimension	32	32

The cell-state encoder linearly maps the G-dimensional control profile to the hidden dimension H, and the drug/dose and response MLPs use H-dimensional hidden layers. The perturbation input concatenates the 1,024-dimensional Morgan fingerprint with one continuous dose coordinate. The graph-refinement modulation map projects the target vector from G to K. The topology readout produces the 64-dimensional prior latent r_d and the gene-wise support pattern s_d defined in the main manuscript. The support-aware gate receives $[r_d; \kappa_d]$, uses a 32-unit hidden layer, and produces a scalar sigmoid output.

Supplementary Table S2. Final DGP-LA optimization settings used for both benchmarks.

Setting	Final value
Optimizer	AdamW ($\beta_1 = 0.9$, $\beta_2 = 0.999$, $\epsilon = 10^{-8}$)
Effective transition-backbone learning rate	2×10^{-5}
Effective graph-prior branch learning rate	1.25×10^{-4}
Learning-rate schedule	constant
Weight decay	5×10^{-4}
Per-GPU / global batch size	32 / 256 (Tahoe); 32 / 128 (NeurIPS)
Maximum optimization steps	8,000 (Tahoe); 5,000 (NeurIPS)
Gradient-norm clipping	10
Random seed	42

S6. Preprocessing, control sampling, and prediction aggregation

For both benchmarks, we used the benchmark-preprocessed, library-size-normalized and log-transformed expression representations. Their supplied expression scale was preserved when constructing the split-specific files; no second normalization or log transformation was applied. The ordered top-2,000 HVG list defined during benchmark preprocessing was fixed across methods and folds. Held-out test objects were projected to this gene order, and HVGs were not recomputed for a held-out test unit.

Control and perturbed cells are not biologically paired one-to-one. During training, each perturbed cell is assigned a basal control input x_i sampled from the control pool with the same cell type and experimental plate. Mini-batches are homogeneous in drug-dose condition. Thus, the matched response set $\mathcal{S}$ and perturbation group $\mathcal{M}$ each collect predicted and observed cells carrying the same drug-dose condition, while allowing different basal control inputs within the set. The sampled control input x_i is the expression-space anchor used for delta alignment, as defined in Supplementary Methods S4.

At test time, control cells from a held-out cell type are used only to supply basal conditional inputs and the control reference required by the prediction task. They do not appear in the training split, and no drug-perturbed cell from a held-out cell type contributes to parameter optimization. For evaluation, predicted and observed cells are separately averaged within each held-out cell type and drug-dose condition to obtain matched pseudobulk profiles. Pearson Delta and Discrimination L1 use signed effects relative to the corresponding control centroids, whereas MSE directly compares the predicted and observed perturbed-expression centroids. Differential expression is computed separately from predicted and observed AnnData objects before Overlap@N is calculated.

S7. Dataset splits and held-out test composition

The same 10 held-out Tahoe cell types were used as test units in both evaluation settings; none served as a validation cell type. Generalization across unseen cell types retained the shared perturbation vocabulary, whereas zero-shot drug perturbation response prediction on unseen cell types additionally imposed mutually disjoint drug identities. Cell counts below include controls.

Supplementary Table S3. Tahoe cell-type split for generalization across unseen cell types.

Split	Number of cell types	Cells
Train	40	66,013,713
Test	10	34,635,077

The held-out test cell types were A-172, LoVo, MIA PaCa-2, A-427, NCI-H2347, NCI-H460, PANC-1, SW480, A498, and HOP62; each contained 1,136–1,137 non-control drug-dose conditions. The processed Tahoe vocabulary contains 379 non-control drugs plus DMSO and 1,137 non-control drug-dose conditions plus control. The three non-control doses are 0.05, 0.5, and 5.0 μM. The perturbation vocabulary is shared across the training cell types and all 10 held-out test cell types in generalization across unseen cell types.

Supplementary Table S4. Tahoe split for zero-shot drug perturbation response prediction on unseen cell types.

Drug partition	Drugs	Drug-dose conditions	Drugs with projected targets
Train	274	822	176 (64.2%)
Test	100	300	67 (67.0%)

The labels in this table denote drug-identity partitions, not cell-type roles; all 10 held-out cell types listed above are test cell types in both evaluation settings. No separate validation drug partition was used in the reported evaluation. The 274 training and 100 test drug identities are mutually disjoint, and their 822 and 300 drug-dose conditions also have zero overlap. Numerical dose levels recur across different drugs, but neither a test drug identity nor any test drug-dose condition appears in training. Zero-shot drug perturbation response prediction

on unseen cell types uses the 374 drugs that have all three dose conditions and at least 50 perturbed cells per condition in every held-out Tahoe cell type. Four of the 379 common non-control drugs lack complete three-dose coverage, and one additional drug fails the minimum held-out-cell criterion. Their identities and condition-level counts are recorded in the released split manifest.

The NeurIPS benchmark contains 240,059 cells across six immune cell types. Four folds rotate T regulatory, T cells CD4+, T cells CD8+, and NK cells as the held-out test cell type; the other three rotating cell types form the training set in each fold. B cells and Myeloid cells are retained as fixed evaluation cell types with fewer non-control treatment compositions.

Supplementary Table S5. NeurIPS held-out cell type folds.

Fold	Held-out test cell type	Training cells	Test cells
1	T regulatory cells	199,691	8,230
2	T cells CD4+	82,950	124,971
3	T cells CD8+	192,361	15,560
4	NK cells	148,761	59,160

T cells CD4+, NK cells, T cells CD8+, and T regulatory cells contain 146, 143, 140, and 141 non-control treatment compositions, respectively. The fixed B-cell and Myeloid evaluation cell types contain 14 and 15 non-control compositions (32,138 cells in total). Each non-control drug has one retained treatment composition: Belinostat is administered at 0.1 μM and the remaining drugs at 1.0 μM.

For zero-shot drug perturbation response prediction on unseen cell types in NeurIPS, 74 training, 30 validation, and 42 test drug identities are mutually disjoint; each corresponds to one retained treatment-composition label in the processed benchmark. The corresponding projected-target coverage is 44/74 (59.5%), 13/30 (43.3%), and 25/42 (59.5%) drugs. The same global drug partition is applied across evaluation on held-out cell types.

S8. Projected PPI and DTI coverage

Coverage was recomputed after projection to the exact ordered 2,000-HVG model space. A mapped node is an HVG present in the full prior gene universe; a connected node has at least one retained non-self PPI edge. Edge counts are unique undirected non-self edges. A drug is prior-active when at least one annotated target remains after HVG projection.

Supplementary Table S6. Prior coverage after projection to 2,000 HVGs.

Quantity	Tahoe	NeurIPS
HVGs mapped to prior universe	1,926	2,000
Connected PPI nodes	1,263	845
Undirected non-self PPI edges	5,374	4,783
Non-control perturbations	379	146
Perturbations with at least one retained target	245 (64.6%)	82 (56.2%)
Perturbations with all-zero projected target vectors	134 (35.4%)	64 (43.8%)

For an all-zero projected target vector, the implementation sets $\Delta h_d = 0$ and $\gamma_d = 0$, reducing the prediction to the data-driven backbone. Main-text Fig. 4a contrasts the full benchmark with the prior-active subset.

S9. Complete metric definitions

The definitions below correspond to `cell-eval` version 0.6.8. Let μ_d^{obs} and μ_d^{pred} denote the observed and predicted pseudobulk centroids for drug-dose condition d, and let $\mu_{\text{ctrl}}^{\text{obs}}$ and $\mu_{\text{ctrl}}^{\text{pred}}$ denote the corresponding control centroids. Define the signed control-referenced effects as $e_d^{\text{obs}} = \mu_d^{\text{obs}} - \mu_{\text{ctrl}}^{\text{obs}}$ and $e_d^{\text{pred}} = \mu_d^{\text{pred}} - \mu_{\text{ctrl}}^{\text{pred}}$. Pearson Delta is

$$\operatorname{corr}\left(e_d^{\text{pred}}, e_d^{\text{obs}}\right), \tag{S12}$$

computed over the 2,000 genes. MSE is $G^{-1}\|\mu_d^{\text{pred}} - \mu_d^{\text{obs}}\|_2^2$.

For Overlap@N, differential expression is computed separately for observed and predicted cells against the matched control with a Wilcoxon test. Genes with Benjamini–Hochberg FDR < 0.05 are ranked by absolute log2 fold change. For condition d, let $\mathcal{G}_d^{\text{obs}}$ be the ordered observed significant-gene list and define the condition-specific value $N_d = |\mathcal{G}_d^{\text{obs}}|$. Let $\text{Top}_{N_d}(\mathcal{G}_d^{\text{pred}})$ be the first N_d predicted significant genes, or all available predicted significant genes when fewer than N_d are available. Then

$$\text{Overlap@N}_d = \frac{\left|\mathcal{G}_d^{\text{obs}} \cap \text{Top}_{N_d}(\mathcal{G}_d^{\text{pred}})\right|}{N_d}. \tag{S13}$$

If a significant-gene rank matrix cannot be formed, or if a condition is absent from either rank matrix, the evaluator returns zero for the affected comparison. Thus, N is not a fixed global cutoff; it is the number of observed significant DE genes for each drug-dose condition.

For Discrimination L1, the signed predicted effect e_d^{pred} is ranked by its L1 distance to every signed observed effect e_j^{obs} over the 2,000-gene expression space. If $r_d \in \{0, \dots, P-1\}$ is the zero-based rank of the matched observed condition among P candidates, then

$$\text{DiscriminationL1}_d = 1 - \frac{r_d}{P}. \tag{S14}$$

Higher scores indicate that the predicted response is more readily identified with its matched observed drug-dose response rather than another candidate response. Reported values average the condition-level metrics within each held-out evaluation unit and then summarize the held-out units.

S10. Systema sensitivity analysis

Systema evaluates perturbation-specific agreement after replacing the control reference with a perturbed-centroid reference and complements this reference-sensitive comparison with centroid accuracy (Viñas Torné et al., 2026). We applied these two metrics to the Tahoe experiment for generalization across unseen cell types. A perturbation condition d denotes one drug-dose combination. Let $\mu_{c,d}^{\text{obs}}$ and $\mu_{c,d}^{\text{pred}}$ be the observed and predicted centroids for condition d in held-out cell type c, and let $\mu_{c,\text{ctrl}}^{\text{obs}}$ be its observed control centroid.

The perturbed-centroid reference was constructed from the mean perturbation effect estimated across training cell types:

$$\bar{e}_{\text{train}} = \frac{1}{|\mathcal{D}_{\text{train}}|} \sum_{d \in \mathcal{D}_{\text{train}}} \frac{1}{|\mathcal{C}_d^{\text{train}}|} \sum_{c' \in \mathcal{C}_d^{\text{train}}} \left(\mu_{c',d}^{\text{obs}} - \mu_{c',\text{ctrl}}^{\text{obs}} \right). \tag{S15}$$

For each held-out cell type, the perturbed-centroid reference is $r_c = \mu_{c,\text{ctrl}}^{\text{obs}} + \bar{e}_{\text{train}}$. Perturbed-centroid Pearson for condition d is then

$$\text{corr}\left(\mu_{c,d}^{\text{pred}} - r_c, \mu_{c,d}^{\text{obs}} - r_c \right). \tag{S16}$$

For each condition, centroid accuracy is the proportion of alternative observed drug-dose centroids in the same held-out cell type that are farther from the predicted centroid than the matched observed centroid:

$$\text{CA}_{c,d} = \frac{1}{|\mathcal{D}_c| - 1} \sum_{q \in \mathcal{D}_c,\, q \neq d} \mathbf{1}\left[\left\| \mu_{c,d}^{\text{pred}} - \mu_{c,d}^{\text{obs}} \right\|_2 < \left\| \mu_{c,d}^{\text{pred}} - \mu_{c,q}^{\text{obs}} \right\|_2 \right]. \tag{S17}$$

Both metrics are computed over the 2,000-gene expression space, averaged over drug-dose conditions within each held-out cell type, and summarized as mean ± s.e.m. across the 10 held-out cell types.

Supplementary Table S7. Systema sensitivity analysis for generalization across unseen cell types on Tahoe.

Method	Perturbed-centroid Pearson	Centroid accuracy
State	0.368 ± 0.023	0.647 ± 0.028
DGP-LA	0.384 ± 0.022	0.662 ± 0.026

DGP-LA improves perturbed-centroid Pearson by 4.4% and centroid accuracy by 2.3% relative to State, with percentage changes calculated from the unrounded means. The gain in perturbed-centroid Pearson shows that the improvement persists after reducing response components shared across perturbations. The centroid-accuracy result provides complementary evidence of improved identification of the matched drug-dose response among alternative responses. Centroid accuracy and Discrimination L1 both assess response identity. The former ranks post-perturbation centroids using Euclidean distance, whereas the latter ranks signed control-referenced effects using L1 distance.

S11. Evaluation on the top 50 DEGs

Supplementary Table S8. Pearson Delta restricted to the top 50 DEGs for generalization across unseen cell types on Tahoe. DEGs are ranked by absolute observed log2 fold change. Values are mean ± s.d. across 10 held-out cell types.

Method	Pearson Delta
CRISP	0.569 ± 0.162
State	0.612 ± 0.106
DGP-LA	0.639 ± 0.111

Supplementary Case Studies

The supplementary case studies extend the main-text gene-level and pathway examples to additional held-out cell-type and drug contexts. They are intended to assess whether the aggregate target-locality and pathway-recovery patterns also appear in individual examples beyond the main-text HepG2/C3A cases.

Supplementary Fig. S1 examines J82. The three panels show separate drug examples, with genes grouped by direct target, one-hop, and two-hop distance in the PPI scaffold. Each panel asks whether graph prior-guided adaptation moves target-neighborhood genes closer to the observed perturbation delta. Across the displayed examples, the full model reduces prediction error for target-proximal genes and improves perturbation-effect correlation, supporting the same locality pattern observed in the aggregate analysis.

Supplementary Fig. S2 repeats the same analysis in LoVo. The three panels again organize selected genes by target-neighborhood distance and compare prediction error relative to the observed perturbation delta. The correction pattern is consistent with graph-derived support acting most visibly near annotated target neighborhoods, rather than producing a uniform expression shift across all selected genes.

Supplementary Fig. S3 evaluates pathway directionality in NCI-H2347 treated with ixazomib citrate. Panel a compares predicted and observed Hallmark NES directions, while panels b and c show representative GSEA running enrichment curves. The full model changes the Hallmark pathway correlation from -0.185 to $+0.806$, indicating improved alignment with observed pathway directions. This example complements the main-text ivermectin case by showing that the latent graph correction can recover coordinated pathway programs in a second cell-type and drug context.

References

A. K. Adduri, D. Gautam, B. Bevilacqua, A. Imran, R. Shah, et al. Predicting cellular responses to perturbation across diverse contexts with State. *bioRxiv*, 2025. doi: 10.1101/2025.06.26.661135.

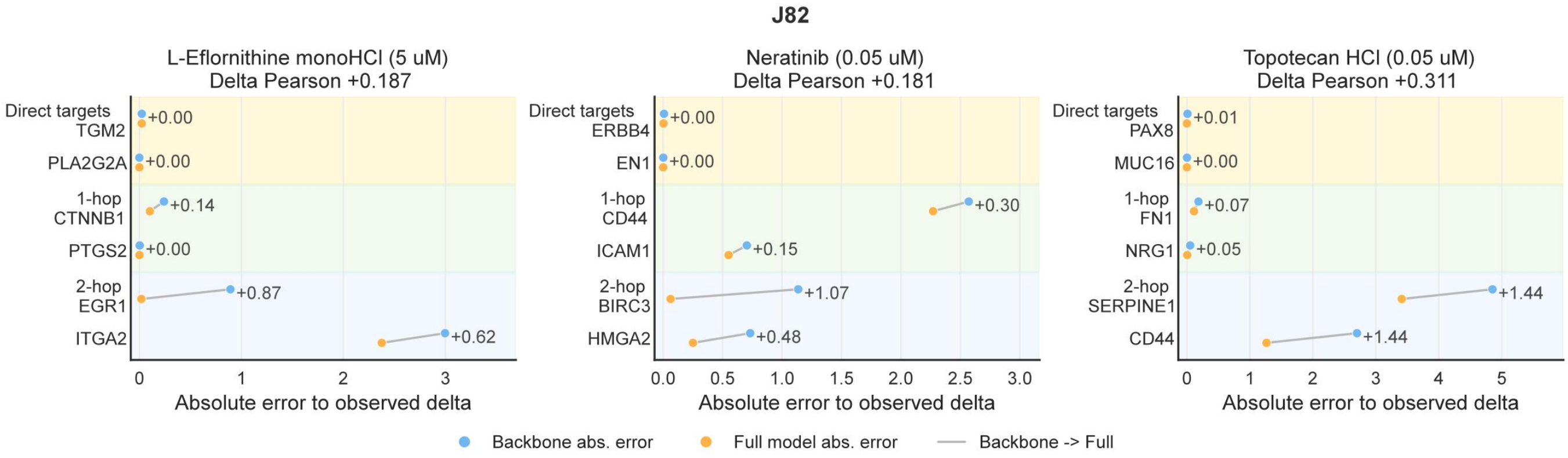

Figure S1: Additional target-neighborhood gene-level case profiles in J82. Three drug examples are shown. Genes are grouped by distance from annotated targets in the PPI scaffold; the horizontal axis reports prediction error relative to the observed perturbation delta for selected genes. The full model reduces error in target-proximal genes and yields higher perturbation-effect correlation.

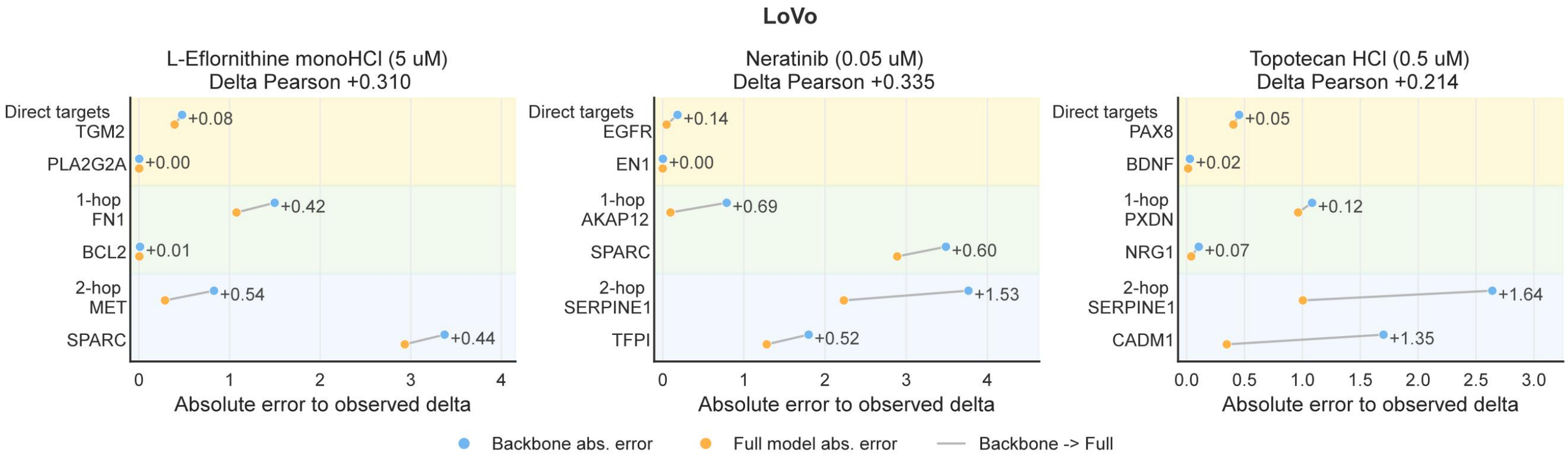

Figure S2: Additional target-neighborhood gene-level case profiles in LoVo. Three drug examples are shown. Genes are grouped by distance from annotated targets in the PPI scaffold; the horizontal axis reports prediction error relative to the observed perturbation delta for selected genes. The full model reduces error in target-proximal genes and yields higher perturbation-effect correlation.

R. Viñas Torné, M. Wiatrak, Z. Piran, S. Fan, L. Jiang, S. A. Teichmann, M. Nitzan, and M. Brbić. Systema: a framework for evaluating genetic perturbation response prediction beyond systematic variation. *Nature Biotechnology*, 44(6):1050–1059, 2026. doi: 10.1038/s41587-025-02777-8.

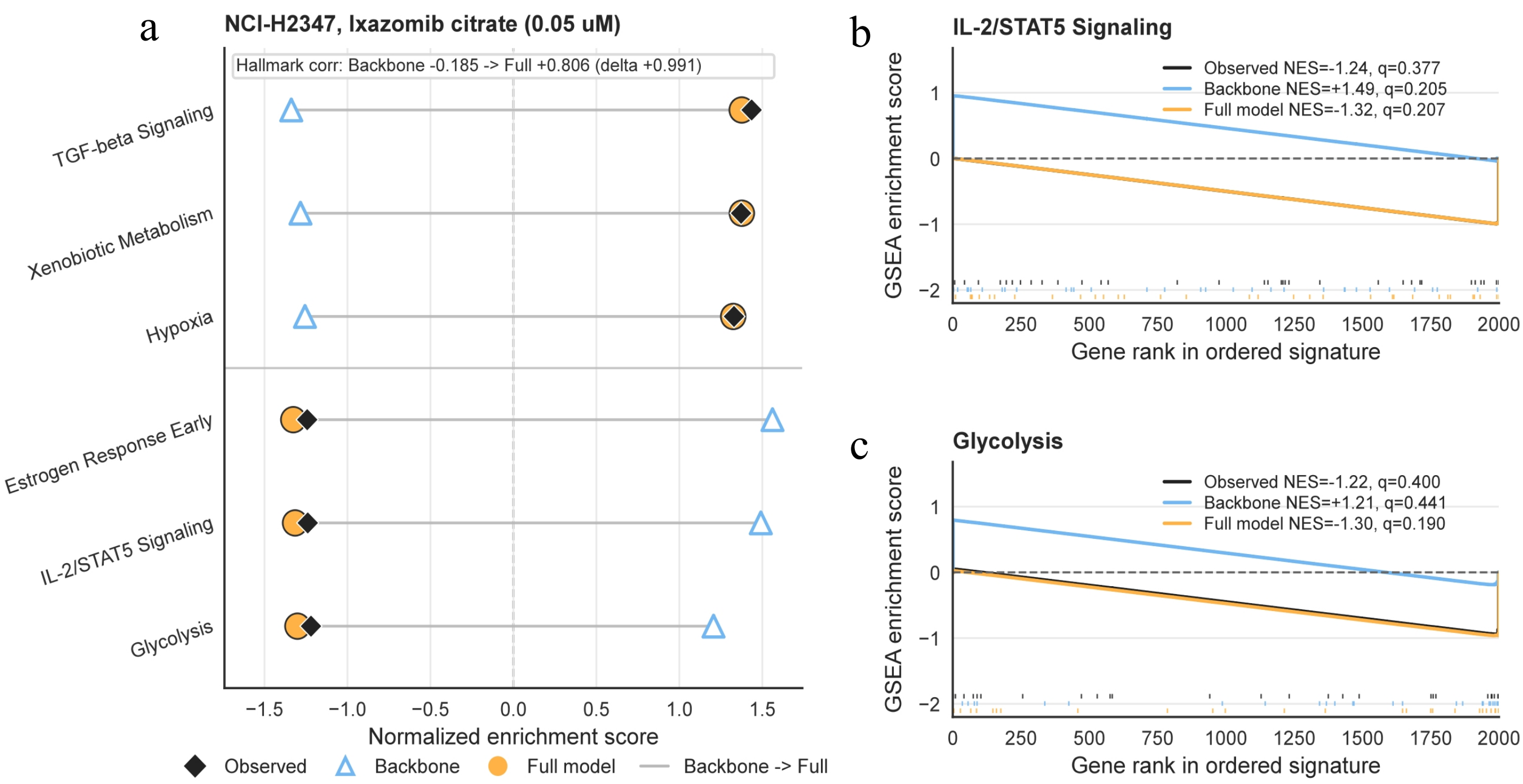

Figure S3: Additional pathway directionality recovery case. a–c, NCI-H2347 treated with ixazomib citrate. a, Hallmark NES directionality. b,c, Representative GSEA running enrichment curves.
